# Image-Guided Focused-Ultrasound CNS Molecular Delivery: An Implementation via Dynamic Contrast-Enhanced Magnetic-Resonance Imaging

**DOI:** 10.1038/s41598-018-22571-8

**Published:** 2018-03-07

**Authors:** Wen-Yen Chai, Po-Chun Chu, Chih-Hung Tsai, Chung-Yin Lin, Hung-Wei Yang, Hsin-Yi Lai, Hao-Li Liu

**Affiliations:** 1 Department of Diagnostic Radiology and Intervention, Chang Gung Memorial Hospital, Taoyuan, Taiwan; 2 Department of Electrical Engineering, Chang Gung University, Taoyuan, Taiwan; 3 Department of Research and Development, NaviFUS Corp, Taipei, Taiwan; 4 Medical Imaging Research Center, Institute for Radiological Research, Chang-Gung University/Chang Gung Memorial Hospital, Taoyuan, Taiwan; 5 Institute of Medical Science and Technology, National Sun Yat-Sen University, Kaohsiung, Taiwan; 6 Interdisciplinary Institute of Neuroscience and Technology (ZIINT), Qiushi Academy for Advanced Studies (QAAS), College of Biomedical Engineering and Instrument Science, Key Laboratory for Biomedical Engineering of Ministry of Education, School of Medicine, Key Laboratory of Medical Neurobiology of Zhejiang Province, Zhejiang University, Hangzhou, Zhejiang People’s Republic of China; 7 Department of Neurosurgery, Chang Gung Memorial Hospital, Taoyuan, Taiwan

## Abstract

Focused ultrasound (FUS) exposure with microbubbles can transiently open the blood-brain barrier (BBB) to deliver therapeutic molecules into CNS tissues. However, delivered molecular distribution/concentration at the target need to be controlled. Dynamic Contrast-Enhanced Magnetic-Resonance Imaging (DCE-MRI) is a well-established protocol for monitoring the pharmacokinetic/pharmacodynamic behavior of FUS-BBB opening. This study investigates the feasibility of using DCE-MRI to estimate molecular CNS penetration under various exposure conditions and molecule sizes. In the 1^st^ stage, a relationship among the imaging index K_trans_, exposure level and molecular size was calibrated and established. In the 2^nd^ stage, various exposure levels and distinct molecules were applied to evaluate the estimated molecular concentration discrepancy with the quantified ones. High correlation (r^2^ = 0.9684) between K_trans_ and transcranial mechanical index (MI) implies K_trans_ can serve as an *in vivo* imaging index to mirror FUS-BBB opening scale. When testing various molecules with the size ranging 1–149 kDa, an overall correlation of r^2^ = 0.9915 between quantified and predicted concentrations was reached, suggesting the established model can provide reasonably accurate estimation. Our work demonstrates the feasibility of estimating molecular penetration through FUS-BBB opening via DCE-MRI and may facilitate development of FUS-induced BBB opening in brain drug delivery.

## Introduction

The blood–brain barrier (BBB) is a highly specialized structure of central nervous system (CNS) blood vessels and capillaries that comprises arachnoid membranes, cerebral capillary endothelial cells, and the choroid plexus epithelium. The BBB protects the normal brain parenchyma from foreign toxic substances because it blocks 98% of molecules weighing in excess of 400 Da^1,2^. But this barrier also prevents the delivery of many potentially effective diagnostic or therapeutic agents, limiting the effectiveness of potential treatments for CNS diseases. Burst-type focused ultrasound (FUS) combined with circulating microbubbles has been verified to increase the permeability of the BBB in a non-invasive, localized, transient and reversible manner^3–6^. In the past decade, the feasibility of FUS-induced BBB opening has been well documented in multiple *in vivo* animal models^3,7–11^ for increasing local concentrations of therapeutic agents for delivery into the CNS. This technology has recently been adopted to be applied clinically to enhance chemotherapeutic agent (Doxorubicin, Cisplatin, or Carboplatin) delivery for human malignant brain tumor treatment, and preliminarily demonstrate its feasibility^12–14^.

To gauge the level of FUS-induced BBB opening, the mechanical index (MI) which is defined as the peak negative acoustic pressure over the square root of the frequency (i.e., MI = P/√f, P in MPa, f in MHz) reflects the scale of inertial cavitation and mechanical bio-effects^15–17^. Previous studies have found a high degree of correlation between the scale of FUS-induced BBB opening and MI using signal intensity (SI) change of contrast-enhanced magnetic resonance imaging (CE-MRI)^3,18^. In addition, MI can serve as an index to identify thresholds (0.46 MI) of FUS-induced BBB opening occurrence^17^, and to indicate adverse effects such as extensive erythrocyte extravasations or brain damage (MI >0.6) after FUS exposure at various central frequencies (i.e., 0.2–2 MHz)^19^. Furthermore, MI is also a useful index to describe microbubble-present acoustic cavitation. In the low MI level (0.41~0.6), the inertial cavitation effect was not detected and the FUS-induced BBB opening was found to rely purely on a stable cavitation effect. Once exposure levels exceeded this range (i.e., MI >0.6), both inertial and stable cavitation are involved in the BBB-opening process^19^. Many studies have discussed the relation between MI and BBB opening, but so far none have addressed the feasibility of using MI as a gauge to measure concentrations of molecular substances with various molecular weights delivered into the brain following FUS-induced BBB opening.

Magnetic resonance imaging (MRI) is one of the most reliable tools for post-operational *in vivo* evaluation of the degree and distribution of BBB opening. In particular, dynamic contrast enhanced MRI (DCE-MRI) has been reported to provide a comprehensive description of dynamic change in FUS-induced BBB opening by calculating the pharmacodynamic (PD) and pharmacokinetic (PK) parameters when administering an MR contrast agent (Gd-DTPA). The MR PD parameter, Gd-based area-under-curve (Gd-AUC) is obtained by accumulating a series of time-dependent Gd-DTPA concentrations and can characterize PD changes of the BBB-opening region^19,20^. Preclinical studies have shown that Gd-AUC is highly correlated with EB-albumin complex accumulation in the brain and has the potential to predict the PD behavior and biodistribution of therapeutic agents^20^. The MR PK parameters, K_trans_ (which describes the influx transfer constant between extracellular extravascular space (EES) and blood plasma) and V_e_ (which describes the EES fractional volume) can describe dynamic change from BBB-opening to BBB-closure. Many studies have verified that the PK parameters can represent the scale of BBB opening and are highly dependent on FUS acoustic pressure^10,21,22^. Furthermore, a high correlation (r^2^ > = 0.7) was found between the PK value and the concentration of dye surrogates or therapeutic agents^10,21^. All evidence points to MR PK/PD parameters from DCE-MRI acquired immediately after FUS exposure as providing accurate predictions of the amount of molecular substances that will be delivered.

While FUS-induced BBB opening has a wide range of applications, preclinical studies have focused on the feasibility testing for various therapeutic agents. Several previous studies have confirmed that FUS exposure allows various drugs to permeate the BBB in increased concentrations and other clinically relevant effects^1,23–26^. For example, FUS-enhanced delivery of liposomal doxorubicin (DOX) was evaluated for glioma treatment^27^. Liposomal-Dox delivery with FUS significantly inhibited tumor growth compared with chemotherapy alone and improved animal survival by nearly 100% in three weekly treatment sessions^28,29^. Other chemotherapeutic agents, such as 1,3-bis (2-chloroethyl)-1-nitrosourea (BCNU), Temozolomide (TMZ), Bevacizumab have also been evaluated. Concentrations of all agents clearly increased in the FUS exposure region and tumor progression was also controlled to improve median survival (Small molecules such as Temozolomide and BCNU reaching 16–72%, and large molecules such as Bevacizumab reaching 135%)^30–32^. However, the impact of tumor treatment is determined by the amount of drug been delivered, which is determined by FUS exposure level and the molecular size of the drug. To date, the relationship of exposure conditions to therapeutic agent size has been largely overlooked and *in-vivo* MRI based predictions of the penetration rates of various molecular substances into the CNS cannot be made without sacrificing the subject animal.

**Figure 1:**
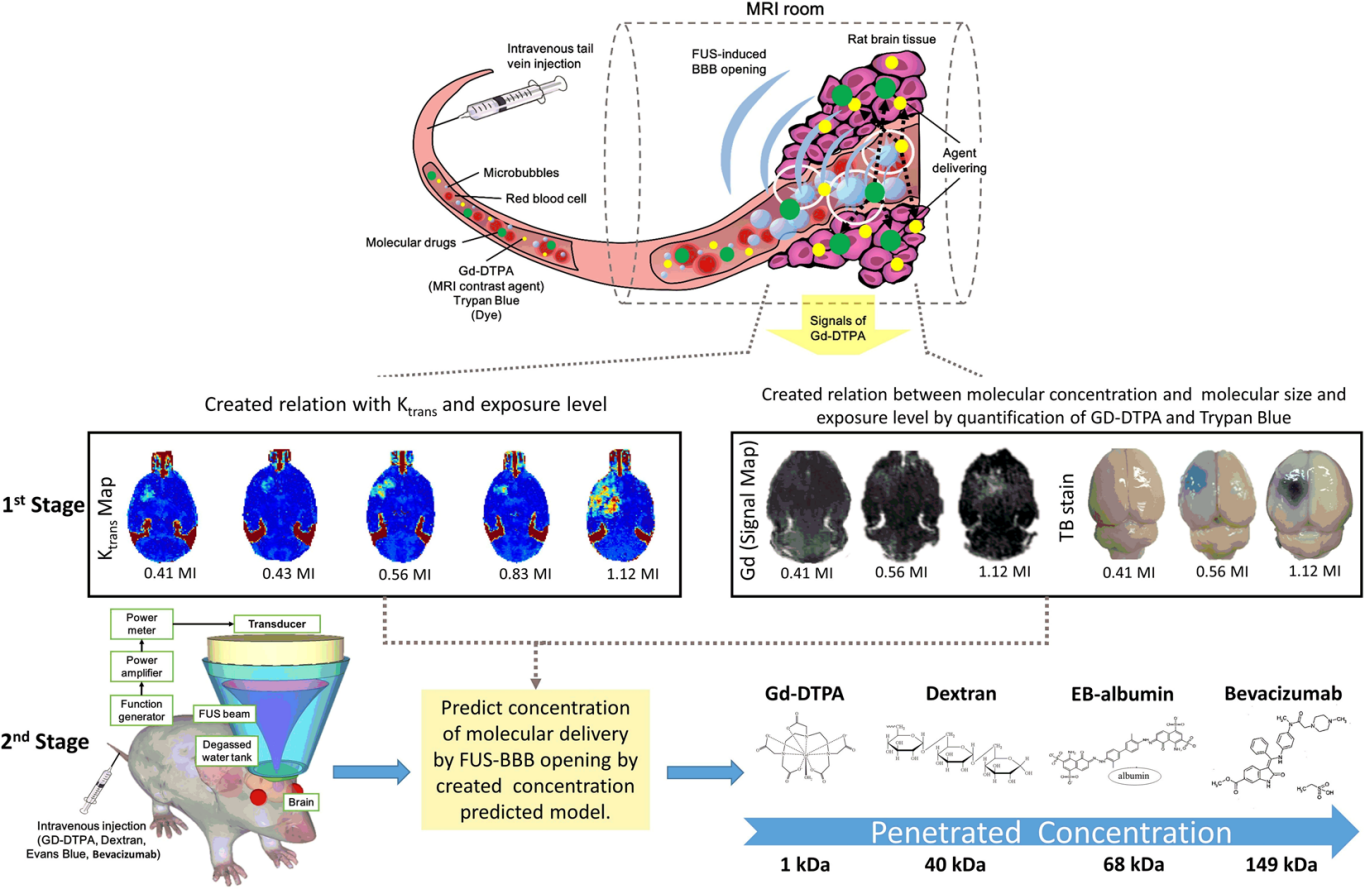
Schematic drawing of proposed two-stage hierarchical structures to demonstrate estimation molecular penetrated concentration by FUS-induced BBB opening via DCE-MRI. In 1^st^ stage, two molecular substances (Gd-DTPA, Trypan blue) were delivered to created two relations and establish penetrated concentration prediction model. In 2^nd^ stage, four molecular substances (Gd-DTPA, Dextran, EB (EB-albumin complex) and Bevacizumab) were delivered and estimated their penetrated concentrations on FUS-induced BBB opening region.

This study investigates the feasibility to establish a CNS drug delivery approach to estimate *in vivo* molecular penetration at various sizes of therapeutic agents based on DCE-MRI. Relationships between DCE-MRI index (K_trans_), ultrasound exposure level (transcranial MI), and delivered molecular concentration (molecules including Gd-DTPA (1 kDa) and Trypan blue-albumin complex (~70 kDa)) was calibrated in the 1^st^ stage. In the 2^nd^ stage, we tested whether 1^st^ stage calibration and established model could be applied to estimate molecular penetration form other distinct molecular substances (Dextran (40 kDa), Evans blue (EB)-albumin complex (~68 kDa), and Bevacizumab (149 kDa)) into rat brain under various FUS exposure level. The estimated molecular concentration was compared with quantification results to verify the accuracy and feasibility of the proposed estimation model. The scheme was summarized and shown in Fig. 1.

## Results

Relationship of FUS-BBB opening degree with various exposure levels (1^st^ Stage).

To investigate the FUS-BBB opening degree and molecular penetration under various FUS settings, we tested FUS with different combinations of exposure frequency (either 0.4 or 1 MHz) and original pressure (0.5–1.25 MI) to produce exposure level in the range of 0.41–1.12 MI (measured by hydrophone for transcranial pressure loss by bone). Details of the animal experiments are summarized in Supplementary Table S1. Animals in group 1–8 received single FUS exposure (10 ms bursts length, 1 Hz pulse pulse-repetition frequency, 90 s exposure duration, 0.2 mL/kg SonoVue®) for evaluation. After FUS exposure, some groups were conducted DCE-MRI with Gd-DTPA injection and other groups were received Trypan blue delivery for quantification.

**Figure 2:**
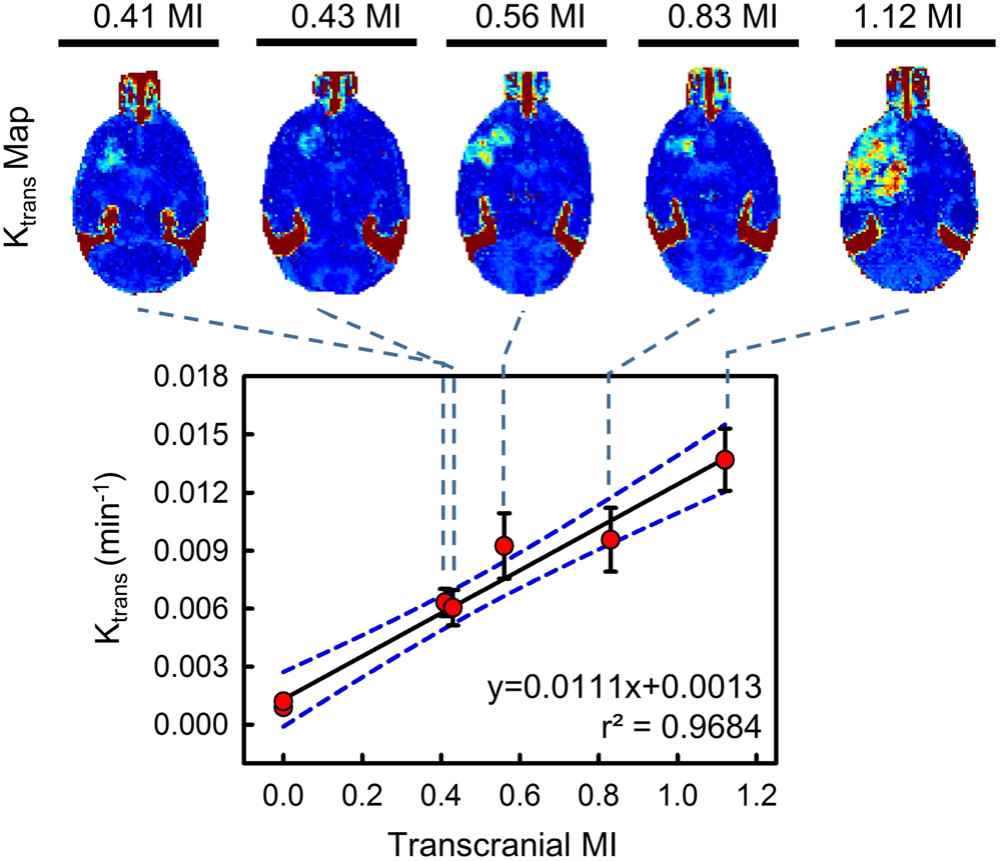
Post-processed K_trans_ maps and correlations of transcranial MI with K_trans_ change within 10 mins. The non-FUS side serves as 0 MI. The K_trans_ was monotonically increased as a function of transcranial MI. A high correlation was found between MI and K_trans_. (r^2^ = 0.9863 in K_trans_).

BBB kinetic change induced by FUS exposure was observed by DCE-MRI image indexs, K_trans_ in Fig. 2 and V_e_ in supplementary Fig. S1. All groups were induced successful BBB-opening with various BBB opening scales under various exposure conditions. The 0.41- and 0.56-MI groups induced mild and intact BBB opening effects with Gd-DTPA leakage, and FUS exposure resulted in increased BBB opening. For the 1.12-MI group, FUS induced more widespread and intense Gd-DTPA leakage than in the former groups. The degree of FUS-induced BBB opening can be clearly observed by K_trans_ mapping. Compared to the contralateral brain (i.e., the non-FUS side), the value clearly increased from a low to high FUS level (K_trans_ level increased from 0.00632 to 0.0137 min^−1^ with MI exposure level from 0.41- to 1.12-MI) and presented a high correlation with MI (r^2^ = 0.9684; the correlation between V_e_ and MI was found to be r^2^ = 0.9333, respectively). Such a high correlation between transcranial MI and K_trans_/V_e_ implies the BBB-opened degree is highly dependent on the actual FUS exposure level. Thus, a relatively higher correlation of K_trans_ was adopted for following analysis, with linear relationship between and transcranial MI can be established as follows:

1 $${\rm{K}}=0.0111\times {\rm{E}}+0.0013$$

where E = ultrasound exposure level (in MI), and K = K_trans_ (in min^−1^). Since the correlation of this linear equation was then used to estimate the transcranial MI for molecular penetration prediction in the 2^nd^ stage.

### Relation establishment of molecular penetration with molecular size and exposure level (1^st^ Stage)

**Figure 3:**
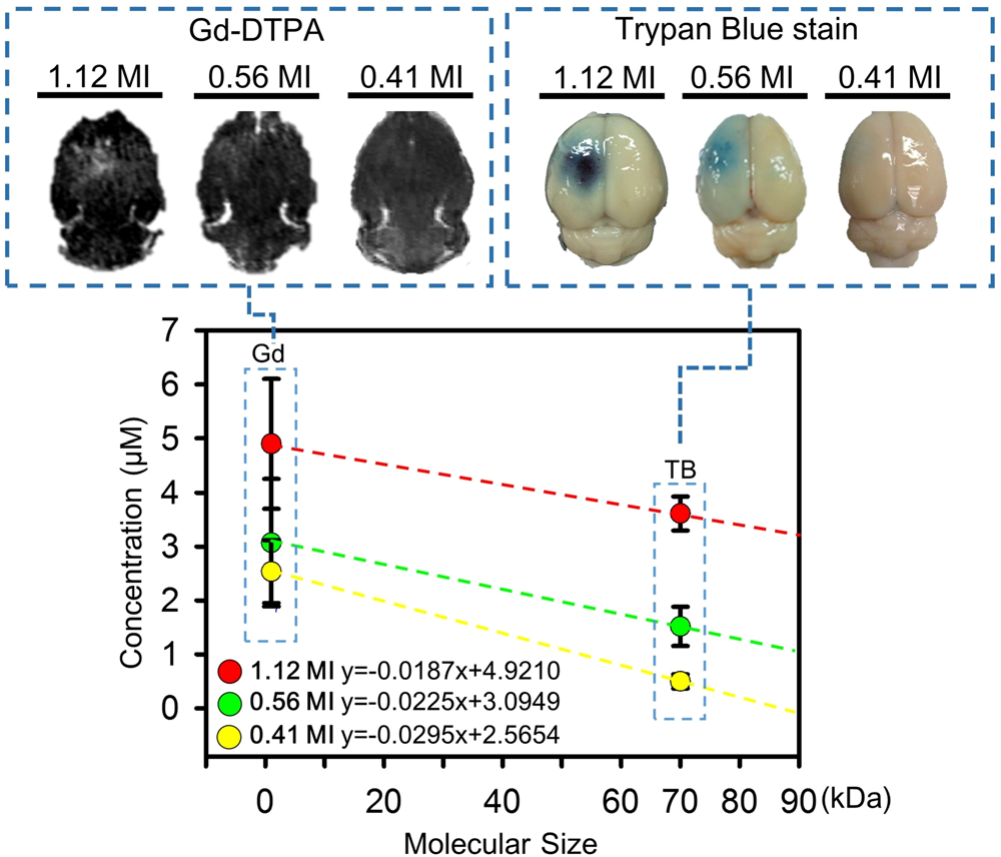
Quantification of different molecular size substances (Gd-DTPA and TB-albumin complex) at different exposure levels (0.41–1.12-MI, after transcranial loss). Increase MI values induced more aggressive BBB-opening with increased Gd leakage in MR signal image and TB leakage in brain. For the same molecular size substances, the penetration concentration increased with MI. For the same MI, the penetration concentration decreased as molecular size increased. Three linearity relations were found between molecular size and concentration at each MI pressure and were combined together to establish new relation (Eq. (2)) by converting the exposure level and molecular size to molecular penetrated concentration.

Two substances, Gd-DTPA and Trypan blue (TB-albumin complex) with molecular sizes of 1 and 70 kDa respectively were delivered to assess the relation of molecular size and penetration under the different FUS exposure conditions (Supplementary Table S1). All animals were sacrificed at 2 hrs after FUS exposure. Figure 3 summarizes the delivery outcomes of these two substances under different exposure conditions. Keeping molecular size constant, higher penetration is achieved with higher MI exposure level (Gd-DTPA increased from 2.45 to 4.9 µM; TB-albumin complex increased from 0.49–3.61 µM when the exposure level was increased 0.41–1.12-MI). This result implies the higher MI can induce larger scale BBB opening to allow increased molecular penetration. Under the same MI, the concentration was monotonically decreased from a small molecular size (Gd-DTPA) to a larger size (TB -albumin complex) which means the molecular penetration efficiency is highly dependent to molecular size. Three linear equations of delivered concentration and molecular size under different MI conditions in Fig. 3 can be used to establish a new prediction model with the established relation by converting the exposure level and molecular size to molecular penetrated concentration:

2 $${\rm{C}}=(0.0129{\rm{E}}-0.0326)\times {\rm{MW}}+(3.3022{\rm{E}}+1.2266)$$

where C is the predicted molecule concentration delivered (in μM), MW is the molecular size (in kDa), and E is the FUS exposure level (in MI). This linear equation was then used to estimate the molecular penetration concentration under a given K_trans_ value and transcranial MI in the 2^nd^ stage.

### Transcranial MI estimation under various delivered substances based on molecular penetration observation (2^nd^ Stage)

**Figure 4:**
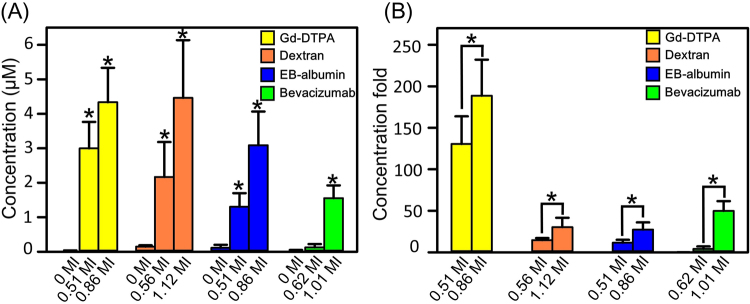
Measured molecular penetration in 2^nd^ stage experiment. (**A**) Penetration concentration (Gd-DTPA, Dextran, EB-albumin complex and bevacizumab) induced by different MI (0.51~1.12MI). For the similar MI (0.51 MI: GD-DTPA, 0.56 MI: Dextran; 0.51 MI: EB-albumin; 0.62 MI: Bevacizumab), penetrated concentration was decreased with lager molecule size. Stars indicate groups with penetrated concentration statistically significant higher than 0 MI group (P < 0.05) (**B**) Penetration concentration fold (compared with 0 MI) of four molecular substances under different MI. For each substance, penetrated concentration fold was increased with higher MI level.

In the 2^nd^ stage, the animals in group 9–19 were conduct to estimate molecular penetration using the predicted modeling established from the 1^st^ stage. Distinct molecules of Dextran (40 kDa), EB-albumin complex (68 kDa), and Bevacizumab (149 kDa) under different of FUS exposure levels were examined (Details of the animal experiments are summarized in Supplementary Table S2). All groups were conducted DCE-MRI scan to obtain K_trans_ maps and the animals were sacrificed for penetrating substance quantification at 2hrs after FUS exposure. An estimated transcranial MI (under the observed K_trans_ level) was then able to be obtained via Eq. (1). In order to test the applicability of this model, various exposure combinations were given (from original MI to estimated transcranial MI): (1) In 0.4 MHz, 0.5–1.25 MI were reduced to 0.41–1.12 MI (groups 9–11, 18–19); (2) In 1 MHz, 0.65 MI/1.25 MI were reduced to 0.51 MI and 0.86 MI (groups 12–15); (3) In 0.5 MHz, 0.62 MI was slightly increase to 0.66MI (groups 16–17). The quantification results of penetration concentration of each substance induced at various estimated transcranial MI were showed in Fig. 4(A). For each molecule in Fig. 4(B), degree of molecular penetration increased (compare with 0 MI) with higher exposure level (GD-DTPA: increased 188 folds at 0.86MI; Dextran: increased 30 folds at 1.12 MI; EB-albumin: increased 27 folds at 0.86 MI; Bevacizumab: increased 49folds at 1.01 MI). For Gd-DTPA at 0.51 MI, Dextran at 0.56 MI, EB-albumin complex at 0.51 MI, bevacizumab at 0.62 MI provided a dramatically concentration decrease as the molecular size increase (Fig. 4(A)). Under similar exposure level, however, bevacizumab provided least penetration which implied that molecular size is a relevant factor for determining molecular penetration efficiency. Detail estimated transcranial MI and quantification results were list in Table 1.

**Table 1:** Summary of the estimated transcranial MI, quantified and predicted concentrations in 2^nd^ stage experiments. Gd = Gd-DTPA, DEX = Dextran, EB = Evans Blue, and BEV = Bevacizumab.

**Freq. (MHz)**	**Type**	**MW (kDa)**	**Orig. MI**	**Est. MI**	**Quantified Con. (μm)**	**Predicted Con. (μm)**	**Error (%)**
1 MHz	Gd	1	1.25	0.86 ± 0.157	4.336 ± 0.998	4.008 ± 0.522	7.55%
1 MHz	Gd	1	0.65	0.51 ± 0.083	2.999 ± 0.776	2.896 ± 0.268	3.41%
1 MHz	EB	68	1.25	0.86 ± 0.157	3.091 ± 0.977	2.675 ± 0.662	13.43%
1 MHz	EB	68	0.65	0.51 ± 0.083	1.303 ± 0.395	1.106 ± 0.343	15.12%
0.5 MHz	Gd	1	0.62	0.66 ± 0.096	3.525 ± 0.462	3.359 ± 0.317	4.69%
0.5 MHz	EB	68	0.62	0.66 ± 0.095	1.649 ± 0.155	1.694 ± 0.569	2.70%
0.4 MHz	DEX	40	1.25	1.12 ± 0.233	4.463 ± 0.315	4.202 ± 0.311	5.86%
0.4 MHz	DEX	40	0.65	0.56 ± 0.152	2.167 ± 0.358	2.062 ± 0.347	4.82%
0.4 MHz	DEX	40	0.50	0.41 ± 0.142	1.601 ± 0.178	1.489 ± 0.332	6.99%
0.4 MHz	BEV	149	1.25	1.01 ± 0.141	1.554 ± 0.369	1.654 ± 0.947	6.42%
0.4 MHz	BEV	149	0.65	0.62 ± 0.115	0.1248 ± 0.148	0 ± 0.6018	—*

^*^The error value can’t be calculated due to the predicted concentration is zero.

### Prediction of various-size molecular concentration under a given exposure level and estimated MI (2^nd^ Stage)

**Figure 5:**
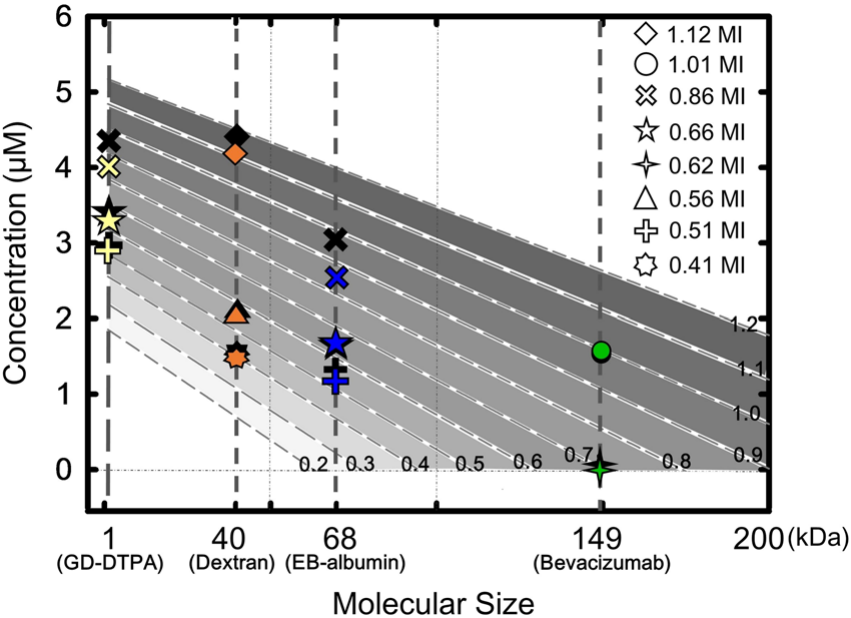
Comparison of quantified and predicted concentrations under different estimated MI pressure values. The sloping dotted line represents the predicted concentration under a specified MI for various molecular sizes. The different level of gray color region in the background is represents the different exposure level region. Different marker shape is represents the estimated transcranial MI via Eq. (1) in 2^nd^ stage group. Colored markers represent the predicted concentration (yellow: Gd-DTPA; orange: Dextran; blue: EB-albumin; green: Bevacizumab) while the black markers represent the quantified concentrations under the estimated exposure MI. The predicted concentrations of Gd-DTPA, Dextran, EB-albumin complex and bevacizumab by transferring estimated MIs and the known molecular size via Eq. (2) were close to the quantified concentrations.

**Figure 6:**
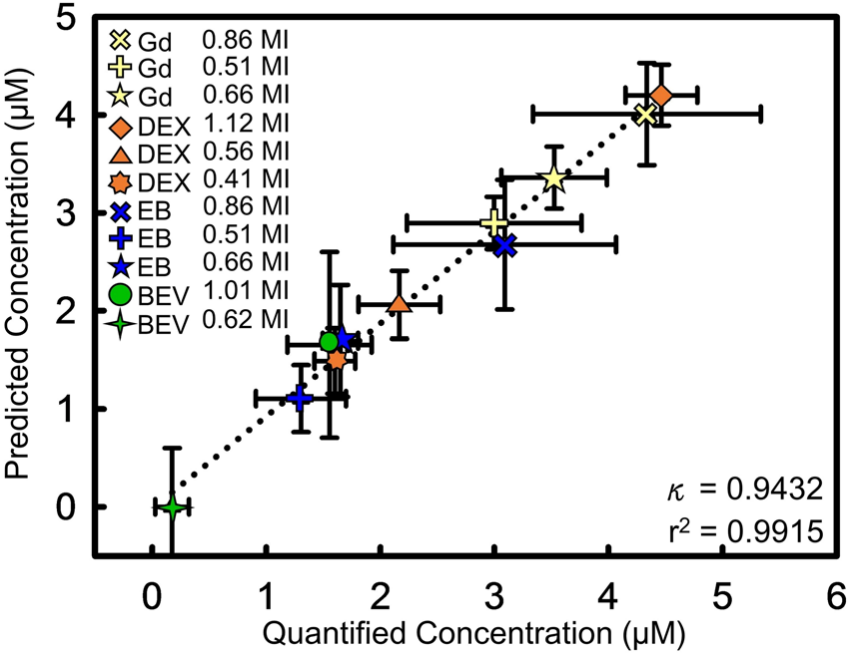
Predicted and actual delivery concentrations of all molecular substances in 2^nd^ stage under different MI. A high correlation was found between the quantified and predicted concentrations (r^2^ = 0.9915).

After knowing the estimated transcranial MI of 9–12 groups and molecular size of delivered substances, the prediction model (Eq. (2)) established in 1^st^ stage was used to estimate various-size molecule penetrated concentration in FUS-induced BBB opening region. Figure 5 compares the predicted and quantified molecular concentrations. The colored markers represent the predicted concentrations under the estimated exposure conditions, whereas the black markers represent the quantified concentrations. The predicted penetration of Gd-DTPA, EB-albumin complex, Dextran induced by 0.86 MI and 1.12 MI were slightly lower than the quantified levels. The predicted Gd-DTPA, Dextran and EB-albumin complex concentration induced by 0.66–0.41 MI nearly matched the quantified ones. The predicted concentration of bevacizumab also matched quantified concentration under 1.01 MI, but was slightly lower under 0.62 MI. Table 1 summarized the quantified and predicted concentration results, showing that prediction error was below 10%, except for that of the EB-albumin complex at a 1-MHz exposure which was 13–15%. Figure 6 showed the correlation between the quantified and predicted concentrations for all tests in 2^nd^ stage. A high correlation between the measured and predicted ones was found (r^2^ = 0.9915) with a κ of nearly 1.

## Discussion

We have demonstrated the feasibility of estimating penetration of various molecular substances following FUS-induced BBB opening via an established imaging index from DCE-MRI. First, K_trans_ can be used to calibrate transcranial MI to eliminate uncertainty of transcranial loss. Second, the penetration concentrations can be accurately estimated by transferring the calibrated transcranial MI via a simple linear transformation. When linking these two results together, molecular penetration induced by FUS exposure can be accurately estimated *in vivo* from an MR image with various ultrasound exposure conditions and various molecular sizes. This approach may facilitate the development of new clinical FUS-induced BBB opening applications to deliver therapeutic molecules for CNS disease treatment.

During FUS exposure, the transcranial ultrasound level will be affect by many uncertain factors such as difference skull thickness^33^, angle of incidence between the FUS beam and the skull surface, and the presence of standing waves within the intact skull cavity^34^. These factors likely add variation to the FUS peak pressure amplitude deposited at the focal point, and result in variance of BBB-opening effect. In our previous study, K_trans_ was found to have the highest correlation among other three MR PK/PD parameters (SI,GD-AUC, Ve) when gauging the BBB opening via transcranial MI^19^. It therefore implies and supports that K_trans_ can serve as a reliable image index to inversely estimate a transcranial MI level reliably, and we have validated this concept via demonstrating a high accuracy in predicting CNS molecular penetration.

In this work, we used wide range of BBB-opened exposure range (ranging from 0.41~1.12 MI) and substances with two different sizes (1, 70 kDa) to establish Eq. (2) which was then applied to estimate concentrations of more diverse substances (up to 149 kDa) during FUS-induced BBB opening. As shown in Fig. 6 and Table 1, the concentrations of Gd-DTPA, Dextran and EB-albumin complex with molecular sizes ranging from 1~68 kDa were predicted accurately (error <16%), along with that of bevacizumab (error <7%) despite its having a molecular size of 149 kDa which exceeds the molecular size range used to formulate Eq. (2). This implies the prediction model (Eq. (2)) can be used to estimate the delivered concentrations of substances measuring greater than 70 kDa in the bound MI range.

Gd-DTPA (1 kDa) has the smallest molecular size used in the proposed concentration predicted model, and clinical drugs with molecular size smaller than 1 kDa may result in less accurate penetration estimation. We have previously delivered 1,3-bis(2-chloroethyl)-1-nitrosourea (BCNU, 214 Da) to into rat brain tissue under 0.36–1.1-MI and the drug concentrations in brain tissue was only about half value when compare with our estimation by our predicted model^30^. In addition, the delivered concentrations of doxorubicin (579 Da) with a BBB permeability ranged from 0.01 to 0.02 min^−1^ at 0.84 MI in Park *et al*. was also fell short of our predicted outcomes^21^. It might be due to: (1) The model establishment and validation falls within the range of 1–149 kDa and hence reliable prediction of extrapolated molecular penetration estimation may not be guaranteed, and (2) Chemotherapeutic agents such as BNCU already preserve good blood-to-brain permeability or doxorubicin exists active efflux transporter pumping function, violating a key assumption made in the proposed model that the molecular influx behavior between the plasma and EES during FUS-BBB opening should be dominant and efflux behavior can be neglected^10^.

**Figure 7:**
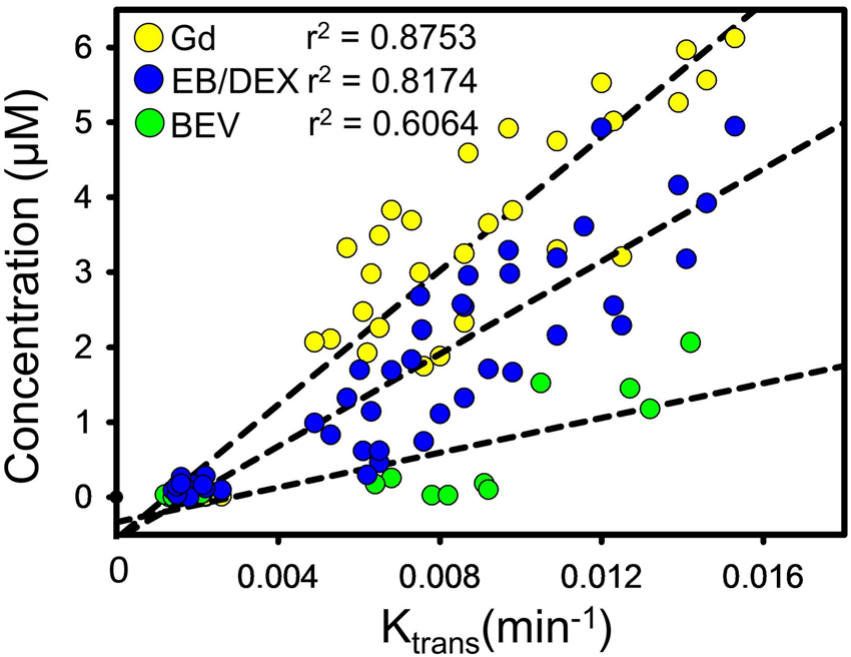
Correlation of K_trans_ and quantified concentration of substances including Gd-DTPA, Dextran/EB-albumin complex, and bevacizumab. Gd-DTPA and bevacizumab respectively had the highest and lowest correlations.

All testing molecules groups were subjected to DCE-MRI and Fig. 7 showed the correlation between the K_trans_ value and quantified concentrations of the delivered substances for each FUS exposure in the 2^nd^ stage. Concentration values substantially increased with K_trans_ level from 0.001 to 0.016 min^−1^. Gd-DTPA had the highest concentrations due to its small molecular size, and also had highest correlation (r^2^ = 0.8753) with K_trans_ value. The Dextran and EB-albumin complexes were grouped together as the molecular sizes of both fall in the middle range of all tested substances, and the Dextran/EB-albumin complexes were found to have an intermediate molecular penetration with a lower correlation (r^2^ = 0.8174) than Gd-DTPA. Bevacizumab had the largest molecular size, and also the lowest molecular penetration and correlation (r^2^ = 0.6064). The bevacizumab concentration began to increase when K_trnas_ exceeded 0.008 ~0.009 min^−1^ (estimated MI is 0.61~0.69 MI), but was otherwise close zero, indicating a 0.61~0.69 MI threshold for bevacizumab to cross through BBB. Below this range, the FUS-induced BBB opening size in the exposure area was too small to allow significant bevacizumab penetration. Chen *et al*. delivered dextran with 4 different molecular sizes (3, 70, 500, 2000 kDa) to assess the BBB opening size by FUS with 3 different MI values by FUS^35^. They reported a BBB opening of up to 70 kDa at 0.4 MI, and larger than 500 kDa when MI increased to 0.69 MI. This result implied a threshold MI for substances with larger molecular sizes, and also explains our finding of the decreased correlation of bevacizumab penetration with K_trans_ when MI is lower than 0.61~0.69 MI (K_trans_ value lower than 0.008~0.009 min^−1^).

The proposed prediction model for estimating molecular penetration is subject to other limitations. The model only considers FUS exposure energy, and neglects other FUS exposure parameters such as burst length, burst repetition rate or microbubble concentration. We also do not evaluate the model’s applicability for variable generalized exposure. However, previous studies have compared three different microbubbles (SonoVue, Defnity, and USphere) in terms of their effect on BBB opening, and was reported to have similar BBB-opening effects and persistence among various types of microbubbles (under a given identical microbubble concentrations)^36^. It implies that microbubble concentration should serve as a dominant factor but not microbubble type, and the proposed prediction approach may be able to apply among microbubble types. Furthermore, the study does not consider the pharmacological properties of the delivered substances such as molecular structure, lipophilicity, solubility, acidity, and absorption. Prior to clinical applications, more experimentation and evaluation is needed to generalize the prediction model, including wider exposure protocols and greater variety of delivery substances.

## Conclusions

This study proposes a CNS drug delivery approach to estimate concentrations of various therapeutic substances delivered via FUS-induced BBB opening. The small error range (<5% for most cases, <16% overall) between quantified and predicted concentrations raise the possibility to applications in medical imaging to monitor and estimate molecular penetration into the CNS during ultrasound-assisted brain drug delivery.

## Materials and Methods

### Preparation of animals

Animal experiments were carried out in accordance with the approved guidelines for the Care and Use of Laboratory Animals. All experimental protocols were approved by the Institutional Animal Care and Use Committee (IACUC) of Chang Gung University and performed according to ARRIVE (Animal Research: Reporting *In Vivo* Experiments) guidelines for the care and use of laboratory animals. A total of 97 animals (male Sprague-Dawley rats, 250–300 g, aged 8 weeks) were randomly assigned into 19 experimental groups. This study assessed the delivery outcomes of molecular substances with five different sizes (Gd-DTPA, 1 kDa; dextran, 40 kDa; Trypan blue (TB)–albumin complex, ~70 kDa; Evans blue (EB)-albumin complex, ~68 kDa; bevacizumab, 149 kDa). Various combinations of exposure frequency and pressure (0.32–0.88 MPa for 0.4-MHz FUS, 0.44 MPa for 0.5 MHz FUS and 0.65–1.25 MPa for 1-MHz FUS) were used to characterize the scale of BBB-opening. The study was performed by two stages: In the 1^st^ stage, groups 1~8 were used to quantitate the two molecular delivery (Gd-DTPA, TB) and establish its relation with DCE-MRI; in the 2^nd^ stage, four molecular delivery (Gd-DTPA, dextran, EB, bevacizumab) was performed on groups 9~19 to evaluate the discrepancy between the estimated molecular concentration and actual measured concentrations. Details of the animal experiments are summarized in Supplementary Tables S1 and S2.

### FUS Instrumentation and exposure

The FUS instrument consists of a function generator (33120 A, Agilent, Palo Alto, CA, USA), a power amplifier (150A100B, Amplifier Research, Souderton, PA, USA) and a 0.4-MHz FUS transducer (Imasonic, France; diameter = 60 mm, radius of curvature = 80 mm), a 0.5-MHz FUS transducer (H104MR; Sonic Concepts, Bothell, WA, USA; diameter = 64 mm, radius of curvature = 63 mm), and a 1-MHz FUS transducer (RK-300, FUS Instruments, Toronto, Ontario, Canada; diameter = 25 mm, radius of curvature = 20 mm). Transducers were measured in a free field filled with deionized/degassed water by a needle type hydrophone. The diameter and length of the half-maximum acoustic pressure of the FUS field were respectively 2.3 and 12 mm for 0.4-MHz FUS, 3 and 8 mm for 0.5-MHz FUS, and 1.2 and 9.8 mm for 1-MHz FUS. The transcranial pressure loss was also measured with an *ex vivo* rat skull placed between the transducer and hydrophone. After transcranial pressure loss measurement in the rat skull, 0.41–1.12 MI were tested to evaluate the association between MI and BBB-opening levels in the 1^st^ stage. This exposure level range covered a sufficiently wide spectrum of known biological and pathological effects of FUS-induced BBB opening from intact BBB-opening to aggressive BBB-opening with erythrocyte extravasations^16,17,30^. All contralateral sides which received only microbubbles were denoted as the non-FUS (MI = 0) group.

All animals were initially anesthetized with 3% isoflurane in 100% O_2_ and continually maintained with 2% isoflurane mixed air during FUS-induced BBB opening. The fur overlying the FUS area was removed for FUS penetration. The animals were placed in a prone position directly under an acrylic water tank with a 4 × 4 cm^2^ window sealed with a thin polyethylene membrane to allow the ultrasound to penetrate through its base. The space between the skull and the window was filled with ultrasound gel and animals received burst-mode FUS at anterior-posterior (AP) 0 mm and midline (ML) −3.5 mm from bregma. Lipid-shell Sulfur hexafluoride (SF6) ultrasound microbubbles (2–5 μm mean diameter 23, 0.2 mL/kg; SonoVue®, Bracco Diagnostics Inc., Milan, Italy) and heparin (0.03 ml/kg; Agglutex, China Chemical and Pharmaceutical Corporation, Taipei, Taiwan) were administered intravenously after dilution with normal saline solution to a total volume of 0.3 ml. Immediately following microbubble injection, burst-mode FUS was delivered with a burst length of 10 ms, pulse-repetition frequency of 1 Hz and duration of 90–120 s. The biological effects induced by this microbubble dosage and FUS pressure have been previously documented^10,20,24,30^. After exposure, substances with different molecular sizes (TB, EB, dextran, and bevacizumab) were administrated intravenously and then DCE-MRI was conducted with the intravenous administration of MRI contrast agent Gd-DTPA (0.3 mL/kg; Magnevist®, Bayer Schering Pharma, Montville, NJ, USA) to obtain pharmacokinetic parameters. Two hours after FUS exposure, all animals were sacrificed and molecule quantification (Gd-DTPA, TB, EB, dextran, bevacizumab) was conducted. The time line for experimental procedures is presented in supplementary Fig. S2.

### Dynamic contrast-enhanced MRI (DCE-MRI)

The *in-vitro* measurements in our previous study, the correlation between spin-lattice relaxivity (R1 = 1/T1) mapping and the Gd-DTPA concentration were all determined using a 7-Tesla MR scanner (Bruker Corp., Billerica, MA, USA)^20^. In some experimental animal groups, the FUS-induced BBB opening was monitored using an MR scanner and a 4-channel surface coil (T7399V3; Bruker Corp., Billerica, MA, USA). Each rat was placed in an acrylic holder, positioned in the center of the magnet, and anesthetized with isoflurane gas (1–2%) at 50–70 breaths/min during the entire MRI procedure.

Following FUS-induced BBB opening, the distribution and dynamics of Gd-DTPA leakage were investigated. Animals were immediately relocated to the MR scanning room to acquire T1-weighted images of DCE-MRI with multiple flip angles. R1 maps and Gd-DTPA concentrations were calculated by transferring these multiple flip angle group images (gradient-recall-echo sequence, TR/TE = 2.31 ms/0.76 ms, slice thickness = 0.8 mm; slice number = 14; matrix = 132 × 192, flip angle = 5°/10°/15°/20°/25°/30°)^10,20^. Following the 20th acquisition, a diluted bolus of Gd-DTPA was IV administrated through a catheter at an infusion rate of 6 mL/min. A series of T1-weighted images were sequentially acquired over a period of 10 min and MRI data were collected for pharmacokinetic analysis by a custom Matlab (Mathworks Inc., Natick, MA, USA) program.

### DCE-MRI for pharmacokinetic analysis

The DCE-MRI PK parameter K_trans_ was obtained to characterize the kinetic behavior of the FUS-induced BBB opening by analyzing a series of Gd-DTPA enhanced T1-weighted images taken over 10 min. Gd-DTPA concentrations were calculated from SI changes of the T1-weighted image, using conversion equations similar to those used in previous study^10^. To calculate the kinetic parameters, the Gd-DTPA concentration curve was fit to the extend Kety model^37–39^ which accounts for the presence of separate extracellular and intravascular compartments. The time-dependent concentration of the contrast agent in tissue can then be described as:

3 $${{\rm{C}}}_{{\rm{t}}}({\rm{t}})={{\rm{v}}}_{{\rm{p}}}{{\rm{C}}}_{{\rm{P}}}({\rm{t}})+{{\rm{K}}}_{{\rm{trans}}}{\int }_{0}{\int }_{0}^{{\rm{t}}}{{\rm{C}}}_{{\rm{p}}}(t\text{'})\times {{\rm{e}}}^{[\frac{-{{\rm{K}}}_{{\rm{trans}}}({\rm{t}}-t\text{'})}{{{\rm{v}}}_{{\rm{e}}}}]}\text{dt}\text{'}$$

where C_p_(t) is the contrast agent concentration in the blood plasma (i.e. the arterial input function (AIF)), C_t_(t) is the contrast concentration in the tissue, K_trans_ is the transfer rate constant from the intravascular system to the EES, and V_p_ and V_e_ are respectively the capillary plasma volume and distribution volume of the contrast agent in the EES (per unit volume of tissue). The SIs of rat brains were converted to C_t_(t) values on the Gd-DTPA concentration time curve, and C_p_(t) was chosen from a region of interest (ROI) in the vein sinus. K_trans_/V_e_ were fitted pixel-by-pixel, using the least squares function in the Matlab optimization toolbox (MathWorks, Inc., Natick, MA, USA) to generate PK parameter maps. A circular ROI was assigned at the BBB opening region to calculate average K_trans_/V_e_ values for the kinetic analysis of BBB opening.

### Gd-DTPA quantification analysis

The T1-weighted images from the DCE-MRI at 10 min following Gd-DTPA IV administration were selected for Gd-DTPA quantification of the BBB opened region. First, spin-lattice relaxivity R1 (=1/T1) maps were calculated by following equation:

4 $$\begin{array}{c}\frac{1}{{{T}}_{1}({t})}=-\frac{1}{{TR}}\times \,\mathrm{log}[\frac{1-{A}}{1-\,\cos \,{\theta }\times {A}}]\\ \,\,\,\,\,{\rm{A}}=\frac{1-exp(-TR/{T}_{10})}{1-\,\cos \,\theta \times exp(-TR/{T}_{10})}\times \frac{S(t)}{S(0)}\end{array}$$

where θ and TR are respectively the flip angle and repetition time of the T1 images, and T_10_ was generated by fitting the signal intensity of pre-contrast T1 images acquired with multiple flip angles. S(t) is the signal intensity of the T1 image over time and S(0) is the signal intensity before the contrast injection. Second, the R1 value and Gd-DTPA concentration were calibrated *in vitro* from our previous study^20^ and the linear relationship is well presented in supplementary Fig. S3(A). Therefore, the Gd-DTPA concentration can be converted from an R1 value by linear transformation for further statistical analysis.

### Trypan blue (TB)- and Evans blue (EB)-albumin complex quantification

The animals in the TB and EB injection group were sacrificed after DCE-MRI for TB- and EB-albumin complex quantification. All animals were first deeply anesthetized with 10% chloral hydrate and infused with heparinized saline through the cardiac ventricle until a colorless infusion fluid was obtained from the atrium. After the rats had been sacrificed by decapitation, the hemispheres of the brain were separated along the transverse suture. Both hemispheres were then weighed and placed in formamide (1 mL/100 mg) at 60 °C for 24 h. The sample was centrifuged for 20 min at 14,000 rpm. The concentration of TB and EB extracted from each brain was determined spectrophotometrically at 595 nm and 620 nm respectively and compared with a standard graph created by recording optical densities from serial dilutions of TB or EB in formamide solutions with blank brain tissues, which were cleared by centrifugation (supplementary Fig. S3(B)). These quantification results were compared with those of molecular substances with different sizes in BBB opening under various FUS energy levels.

### Dextran and bevacizumab quantification

The animals in the dextran injection group were sacrificed after FUS exposure and all brain tissue samples were analyzed using high performance liquid chromatography (HPLC) with a UV detector (L-2400; Hitachi, Tokyo, Japan), a pump (L-2130; Hitachi, Tokyo, Japan), and a 4.6 × 250-mm C-18 column (SUPELCOSIL). The mobile phase was an acetonitrile–water (46:54, v/v) mixture delivered at a flow rate of 1 mL/min at 25 °C and with a measuring wavelength of 335 nm. Dextran standard solutions (0.1–0.8 mg/mL) were prepared and 10 µL of the standard solutions were analyzed using HPLC to establish a standard curve (retention time, 11.2 min). Brain homogenates were transferred to a 15 mL conical centrifuge tube and the Dextran was extracted with acetonitrile by means of 30 min sonication at 10 °C. After centrifugation, the supernatant was reserved and the pellet was re-extracted twice with 1 mL of acetonitrile. The combined supernatants were filtered (0.22 µm), extracted with 1 mL acetonitrile, and diluted with acetonitrile to a final volume of 4 mL. 10 µL of each brain extract were analyzed by using HPLC and the dextran concentrations calculated from the standard curve. The animals in the bevacizumab injection group were sacrificed after FUS exposure and DCE-MRI. The extracted bevacizumab was analyzed with HPLC as previously described^32^. Supplementary Fig. S3(C,D) respectively show the standard curves of dextran and bevacizumab.

### Statistical analysis

Statistical analysis was performed using SPSS 20.0 software (IBM SPSS statistics; IBM Corp., Armonk, NY, USA) by two researchers blind to animal assignment. The DCE-MRI parameter data (K_trans_) and molecular substance quantification data are presented as mean ± standard deviation of the mean and analyzed by one-way ANOVA. Differences were considered to be statistically significant when p < 0.05. The coefficients of Eqs (1) and (2) with 95% confidence intervals (CI) were found using linear least squares regression. Additional analyses included least-squares linear regression and calculation of correlation coefficients for data comparison.

## Electronic supplementary material

Supplementary information
