# Supplementary material for: Image-Guided Focused-Ultrasound CNS Molecular Delivery: An Implementation via Dynamic Contrast-Enhanced Magnetic-Resonance Imaging
Source: Sci Rep. 2018 Mar 7;8:4151. doi: 10.1038/s41598-018-22571-8 (PMC5841286; doi:10.1038/s41598-018-22571-8)
Supplement: Supplementary file 1 — Supplementary information [file 41598_2018_22571_MOESM1_ESM.docx]

**Supplementary Information**

**Image-Guided Focused-Ultrasound CNS Molecular Delivery: An Implementation via Dynamic Contrast-Enhanced Magnetic-Resonance Imaging**

Wen-Yen Chai^1,2^, Po-Chun Chu^3^, Chih-Hung Tsai^2^, Chung-Yin Lin^4^, Hung-Wei Yang^5^, Hsin-Yi Lai^6^, Hao-Li Liu^2,7*^

^1^Department of Diagnostic Radiology and Intervention, Chang Gung Memorial Hospital, Taoyuan, Taiwan

^2^Department of Electrical Engineering, Chang Gung University, Taoyuan, Taiwan

^3^Department of Research and Development, NaviFUS Corp.,Taipei, Taiwan

^4^Medical Imaging Research Center, Institute for Radiological Research, Chang-Gung University/Chang Gung Memorial Hospital, Taoyuan, Taiwan

^5^Institute of Medical Science and Technology, National Sun Yat-Sen University, Kaohsiung, Taiwan

^6^Institute of Neuroscience and Technology, Zhejiang University, Zhejiang, People's Republic of China

^7^Department of Neurosurgery, Chang Gung Memorial Hospital, Taoyuan, Taiwan.


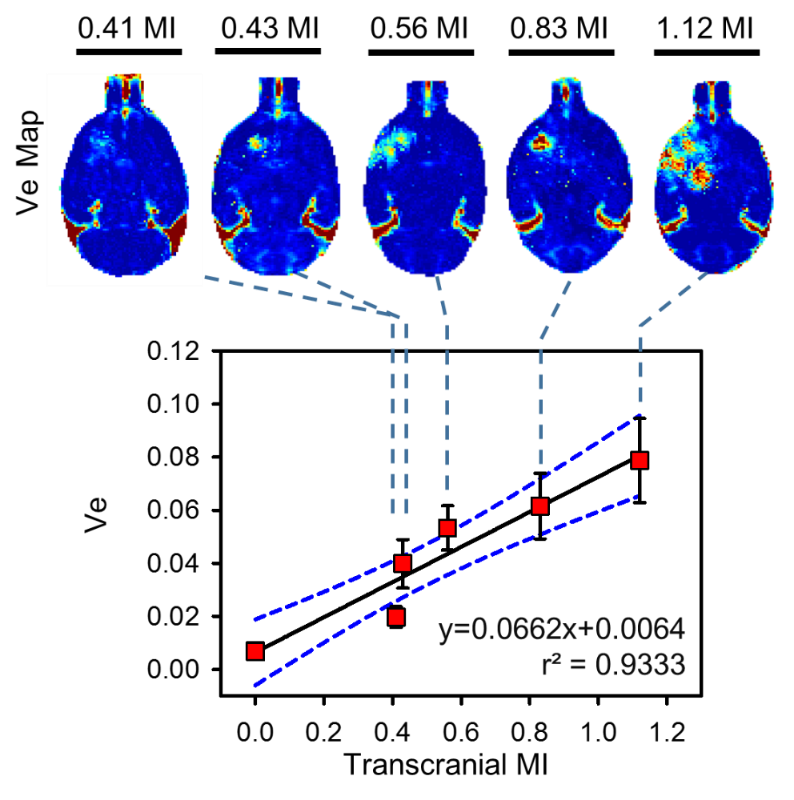


**Figure S1. Post-processed V_e_ maps and its correlations with transcranial MI.** The non-FUS side serves as 0 MI. The V_e_ was monotonically increased as a function of transcranial MI. A high correlation was found between MI and V_e_. (r^2^=0.9333). When comparing with Fig. 2, the correlation of V_e_ and MI was found to be comparable to the correlation of K_trans_ and MI (r^2^=0.9684).

**
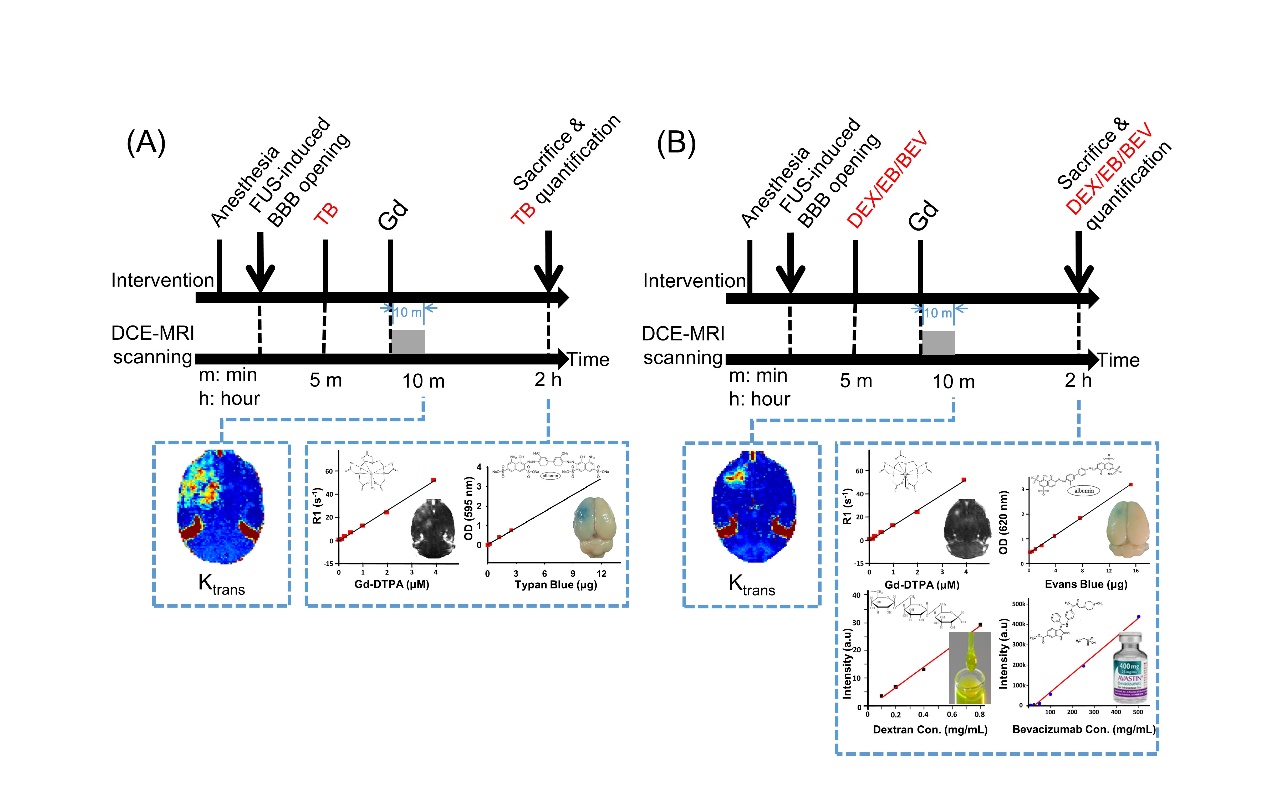
**

**Figure S2. Summary of the time line for experimental animal groups.** (A) In 1^st^ stage, Gd (Gd-DTPA) and TB (Trypan blue) were delivered to establish the prediction model of molecular penetration after FUS-induced BBB opening. (B) In 2^nd^ stage, Gd, DEX= Dextran, EB=Evans Blue, and BEV= Bevacizumab were delivered and quantified 2hrs after FUS exposure. Quantification results of these molecular substances will compare with estimated concentration by proposed concentration predicted model.

**
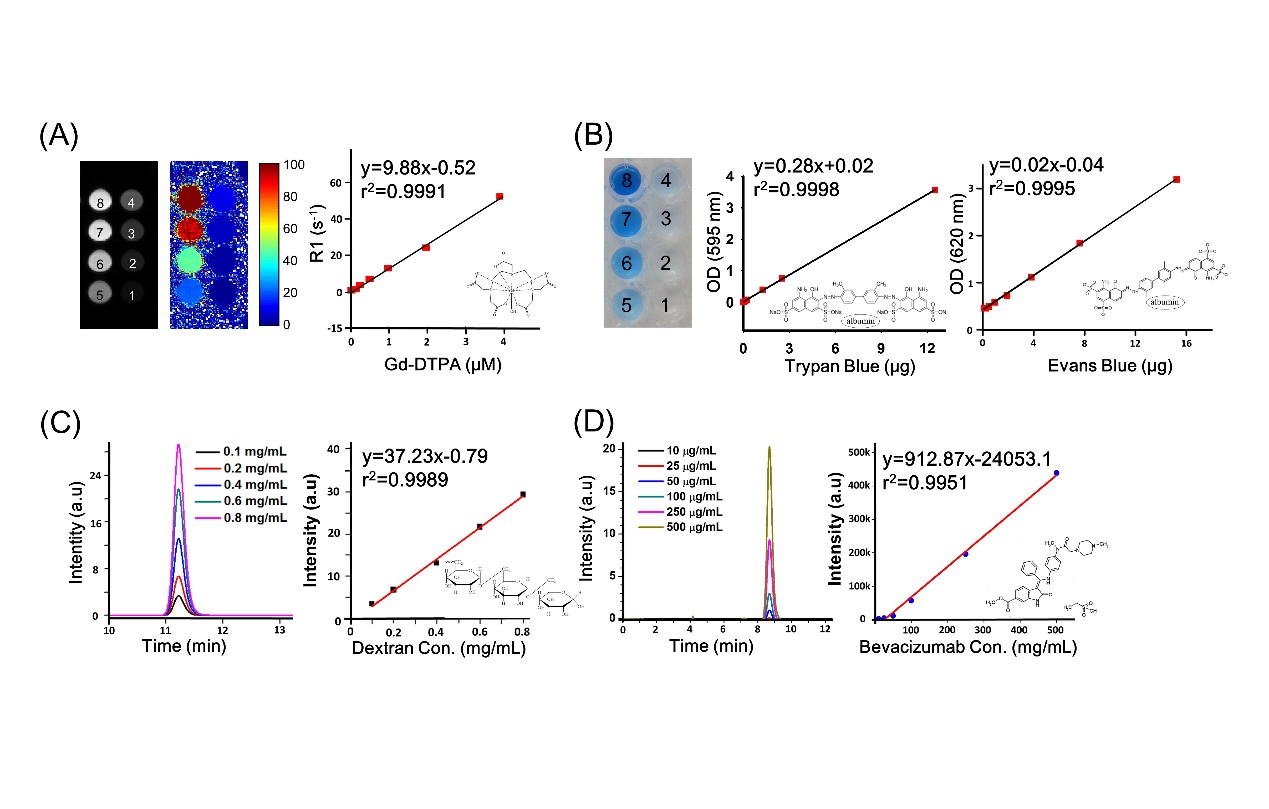
**

**Figure S3. Different quantification methods for the delivery of five molecular substances.** (A, B) GD, TB, EB content were quantified using the linear regression standard curve derived from eight different concentrations. (C, D) Dextran or bevacizumab were also quantified by calibration of the standard curve from HPLC.

**Table S1.** **Experimental design in 1^st^ stage. Two frequency transducers (0.4MHz, 1MHz) with three difference exposure pressures combinations were used in FUS-induced BBB opening.** Total 48 animals with two delivered molecular substances (Gd-DTPA, Trypan blue) were separated into each of the 0.4- and 1-MHz groups. MI: mechanical index; n: animal number in group.

| **Group  (n)** | **Frequency (MHz)** | **Original Exposure level (MI)** | **Delivered Molecule** |
| --- | --- | --- | --- |
| 1 (6) | 0.4 | 0.50 | Gd-DTPA |
| 2 (6) | 0.4 | 0.50 | Trypan blue |
| 3 (6) | 0.4 | 0.65 | Gd-DTPA |
| 4 (6) | 0.4 | 0.65 | Trypan blue |
| 5 (6) | 0.4 | 1.25 | Gd-DTPA |
| 6 (6) | 0.4 | 1.25 | Trypan blue |
| 7 (6) | 1.0 | 0.65 | Gd-DTPA |
| 8 (6) | 1.0 | 1.25 | Gd-DTPA |

**Table S2**. **Experimental design in 2^nd^ stage. Three frequency transducers (0.4MHz, 0.5MHz, 1MHz) with four difference exposure pressures combinations were used in FUS-induced BBB opening.** Total 49 animals with four delivered molecular substances (Gd-DTPA, Dextran, Evans blue, Bevacizumab) were separated into each of the 0.4-, 0.5- and 1-MHz groups. MI: mechanical index; n: animal number in group.

| **Group (n)** | **Frequency (MHz)** | **Original Exposure level (MI)** | **Delivered Molecule** |
| --- | --- | --- | --- |
| 9 (3) | 0.4 | 0.50 | Dextran |
| 10 (3) | 0.4 | 0.65 | Dextran |
| 11 (3) | 0.4 | 1.25 | Dextran |
| 12 (6) | 1.0 | 0.65 | Gd-DTPA |
| 13 (6) | 1.0 | 0.65 | Evans blue |
| 14 (6) | 1.0 | 1.25 | Gd-DTPA |
| 15 (6) | 1.0 | 1.25 | Evans blue |
| 16 (3) | 0.5 | 0.62 | Gd-DTPA |
| 17 (3) | 0.5 | 0.62 | Evans blue |
| 18 (6) | 0.4 | 0.65 | Bevacizumab |
| 19 (4) | 0.4 | 1.25 | Bevacizumab |
